# Transthyretin Cardiac Amyloidosis: Current and Emerging Therapies

**DOI:** 10.1007/s11886-024-02172-w

**Published:** 2025-01-22

**Authors:** Aditi G. M. Patel, Pengyang Li, Narotham Badrish, Aditya Kesari, Keyur B. Shah

**Affiliations:** The Pauley Heart Center, Virginia Commonwealth University, 1200 East Broad Street West Hospital, 8th Floor, West Wing, Richmond, VA 23231 USA

**Keywords:** Amyloidosis, Transthyretin, Cardiomyopathy, Heart failure, Therapy, Treatment

## Abstract

**Purpose of Review:**

In this article, we describe current and newer TTR stabilizers, TTR silencers which include small interfering RNA agents (siRNA), antisense oligonucleotides (ASO) and CRISPR-Cas9 gene editing, and TTR depleters, which investigates the use of monoclonal antibodies to remove amyloid fibril deposits for patients with advanced disease.

**Recent Findings:**

Once thought to be a rare and fatal condition, increased recognition, improved non-invasive diagnostic tools, and the explosive development of novel therapies, has transformed the landscape of transthyretin amyloid cardiomyopathy (ATTR-CM). Advances in cardiac imaging with respect to echocardiography, cardiac magnetic resonance imaging (CMR), and radionuclide bone scintigraphy has increased the diagnosis of ATTR-CM over the last twenty years. Ongoing clinical trials are evaluating several novel therapies at several mechanistic targets in the transthyretin (TTR) amyloidogenesis cascade, including the recently published findings from the study of vutrisiran, a siRNA agent.

**Summary:**

Our review provides a comprehensive summary of current and emerging therapies for ATTR-CM. While these are promising, disease-modifying treatments, reaching vulnerable populations early in the disease course should be a focus for future studies and interventions.

## Introduction

Amyloidosis is a systemic disease arising from misfolded proteins and fibrillogenesis causing amyloid deposits and resulting in end-organ destruction. There are more than 40 different types of precursor proteins which can breakdown to form amyloid fibril deposits [1, 2]. One of the most commonly found proteins that affect the heart disease is transthyretin (TTR). TTR-related amyloidosis (ATTR) is further categorized into a wild-type (wtATTR) (spontaneously acquired with aging) or hereditary variants (hATTR) [1–5].

In this issue of *Current Cardiology Reports*, we will review the latest advances in drug development and clinical application for clinicians managing transthyretin cardiac amyloidosis. This review is of current drugs approved or being studied for ATTR cardiomyopathy (ATTR-CM), including stabilizers, silencers, and depleters.

Previously, liver transplantation and symptom management were the mainstay of treatment for hATTR, and there were no disease-specific therapies for ATTR-CM [1–4, 6]. In carefully selected patients there would have been consideration for heart or combined heart-liver transplantation with excellent outcomes [6–8].

The first TTR stabilizers, such as diflunisal and tafamidis, were studied in the mid-2000s [9–12]. Tafamidis was approved for ATTR in Europe in 2011 and would later be approved with ATTR-CM in 2019 in the United States [12, 13]. With burgeoning awareness and improved diagnostics, interest in clinical research surrounding ATTR-CM is rapidly accelerating. Ongoing studies not only include additional TTR stabilizers [14] but also silencers, and depleters [6, 15–21].

## TTR Stabilizers

Currently, the standard of care for the treatment of ATTR-CM is a class of medication known as TTR stabilizers (Table 1). Their role is to bind to the TTR tetramer and prevent the formation of amyloid either by inhibiting dissociation or by increasing structural stability.

**Table 1:** TTR stabilizing therapies for transthyretin amyloid cardiomyopathy (ATTR-CM)

Drug	Clinical trial (year)	Design	Population	% Black patients	V142I patients	Outcomes
Non-Selective						
Diflunisal	Pilot Study[11]	single-arm, open-label study; oral diflunisal 250 mg BID with mean follow-up of 0.9 ± 0.3 years	*N* = 13 (*n* = 7 wtATTR, *n* = 6 hATTR)	2% (*n* = 2)	Not reported	Safe. No significant change in cardiac structure, function, or biomarkers.
Selective						
Tafamidis*	ATTR-ACT[12]	Phase 3, multicenter, randomized, 2:1:2 ratio, double-blind, placebo-controlled to receive tafamidis 80 mg, 20 mg or placebo with 30 months follow-up	*N* = 441 (*n* = 106 hATTR, *n* = 335 wtATTR)	14% (*n* = 63)	Not reported	Lower all-cause mortality and frequency of CV-related hospitalizations. increased 6MWT, KCCQ-OS score
Acoramidis* (formerly known as AG10)	ATTRibute-CM [14]	Phase 3 clinical, randomized, 2:1 ratio, double-blind, placebo controlled to receive acoramidis 800 mg BID or placebo for 30 months	*N* = 632 (*n* = 61 hATTR, *n* = 571 wtATTR)	4.7% (*n* = 30)	59% (*n* = 36)	Lower all-cause mortality and frequency of CV-related hospitalization, similar incidence in adverse events compared to placebo group

(Table 1 partially adapted from: Tomasoni D, et al. Front. Cardiovasc. Med., vol. 10, May 2023:10:1154594. 10.3389/fcvm.2023.1154594; Creative Commons user license https://creativecommons.org/licenses/by/4.0/) [2]

* Inclusion criteria: patients aged 18–90 diagnosed with hereditary or wild type transthyretin amyloidosis confirmed by biopsy (e.g. endomyocardial, abdominal fat pad, gastrointestinal, bone marrow, tissue), with mass spectrometry or immmunohistochemical analysis or scintigraphy and biochemical analysis for exclusion of light chain (AL) amyloidosis

### Diflunisal

Diflunisal is a non-steroidal anti-inflammatory drug (NSAID) synthesized from 2,4-difluoroaniline that ties to the T_4_ binding site of the transthyretin tetramer. This in turn inhibits dissociation into monomers and inhibits aggregate formation [22].

There have been several retrospective analyses that have shown that diflunisal can halt disease progression and improve quality of life within the follow up time of 12–24 months [9, 10, 22]. Although its potency is less than tafamidis, diflunisal is an effective kinetic stabilizer due to having good bioavailability and drug half-life with lack of substantial plasma metabolites [23].

One retrospective analysis of 81 patients showed that after 1 year of diflunisal administration, there were measurable differences in some parameters of cardiac structure including global longitudinal strain [10]. In a retrospective cohort analysis of wtATTR patients with cardiomyopathy, diflunisal administration was associated with improved survival and overall stability in clinical markers (troponin I and B-type natriuretic peptide (BNP)) and echocardiographic measurements (intraventricular septal thickness and left ventricular (LV) ejection fraction (EF)) [24]. In a follow-up single-arm, open label study, diflunisal was found to be safe and showed no statistically significant difference in cardiac structure, function or cardiac biomarkers, suggestive of limited progression of ATTR-CM disease of patients on diflunisal [11].

These studies show diflunisal can be a treatment option in patients who are otherwise ineligible for other approved therapies. However, diflunisal is an NSAID; it can have side effects since as fluid retention, HTN, and renal toxicity especially in older adults. This limits its efficacy and restricts the patient population that can safely take this medication [23].

### Tafamidis

Tafamidis is an orally bioavailable derivative that acts similarly to diflunisal by binding to the T4 binding site of the transthyretin tetramer, but with a higher affinity [1]. It is the first disease-modifying therapy to be approved for treating adults with both wtATTR-CM and hATTR-CM.

The Safety and Efficacy of Tafamidis in Patients With Transthreytin Cardiomyopathy (ATTR-ACT; NCT01994889) was the first clinical trial that showed tafamidis was associated with a lower all-cause mortality in patients with ATTR amyloidosis [12]. This study randomized patients in a 2:1: ratio to receive 80 mg tafamidis, 20 mg tafamidis, or placebo for 30 months. Both wtATTR-CM and hATTR-CM patients were included in the trial. The study also demonstrated a lower rate of cardiovascular hospitalizations, as well as lower rate of decline in a 6 min walking test and in quality of life. The effect of tafamidis was also dose-dependent, showing greater mean TTR concentrations in the 80 mg tafamidis group compared to the 20 mg group [25]. There was also a long-term extension study which had a mean follow up of 58.5 months. This analysis demonstrated continued benefits of tafamidis treatment, including better survival independent of New York Heart Association (NYHA) class and genotype compared to the placebo [26]. Of note, NYHA class IV patients were excluded from this trial. The NYHA class I or II subgroups showed a 44% decrease, and the NYHA class III group had a 35% decrease in risk of all-cause mortality. This trial used tafamidis meglumine, and subsequently another single center, phase I trial established equivalence in bioavailability of tafamidis 61 mg free-acid capsules (Vyndamax) when compared to 80 mg tafamidis meglumine (Vyndaqel) [27]. A post hoc analysis showed that tafamidis treatment was associated with a significantly smaller decline in kidney function and a higher likelihood of improvement in CKD and eGFR [28]. Another post hoc analysis exploring treatment efficacy in octogenarian patients also demonstrated efficacy with tafamidis treatment in elderly populations [29].

Tafamidis treatment also yielded significant changes notable on cardiac imaging as well. A prospective analysis on the effects of tafamidis treatment in 12 months showed preserved cardiac magnetic resonance (CMR)-measured biventricular function and reduced left ventricular (LV) mass compared to the placebo group [30]. Irrespective of tafamidis treatment, extracellular volume and T1-mapping did not significantly change from baseline in both the tafamidis and placebo groups. Another post hoc analysis demonstrated that tafamidis slowed the decline in LV systolic and diastolic function as measured by echocardiography [13].

A long-term extension (LTE) study for the patients in the ATTR-ACT trial was conducted in which patients in both the tafamidis and placebo group would be given tafamidis [31]. All-cause mortality was higher in patients with LVEF < 50%, but those treated continuously with tafamidis had a 47% reduction in mortality risk compared to those who received delayed treatment, irrespective of ejection fraction.

### Acoramidis

Acoramidis, formerly known as AG10, is a stabilizer molecule that acts to inhibit dissociation of tetrameric TTR and ex vivo has been shown to have more than 90% stabilization of the TTR molecule [32]. In vitro studies show superior selectivity and efficacy towards stabilization of TTR compared to tafamidis and diflunisal [32]. In the patients who completed the phase 2 study and enrolled in the open-label extension (*n* = 25), acoramidis was well-tolerated with adverse events that were consistent with disease severity, concurrent illness, and/or age [33]. They demonstrated stable N-terminal, pro-B-type natriuretic peptide (NT-proBNP) and sustained increases in serum TTR.

The Efficacy and Safety of AG10 in Subjects With Transthyretin Amyloid Cardiomyopathy (ATTRibute-CM; NCT03860935) was a phase 3 trial which was designed to evaluate the safety and efficacy of acoramidis in patients with either wtATTR-CM (*n* = 571) or hATTR-CM (*n* = 61) [14]. Patients were treated with either acoramidis 800 mg twice a day or placebo. The study evaluated 4 outcomes including reduction in all-cause mortality, hospitalization for cardiovascular causes, change in baseline of NT-proBNP and change in baseline of distance in 6-minute walk test (6MWT). In the 632 patients that underwent randomization, the primary analysis favored acoramidis over placebo (*P* < 0.001) with a corresponding win ratio of 1.8. In the 30-month follow up, the use of acoramidis demonstrated significantly better four-step primary hierarchical outcome compared to placebo and demonstrated similar rate of adverse events between the 2 groups with less frequent serious adverse events in the acoramidis group [14]. It is currently pending FDA approval for use in patients with ATTR-CM.

The upcoming Acoramidis Transthyretin Amyloidosis Prevention Trial in the Young (ACT-EARLY) Study in Asymptomatic Carriers of a Pathogenic TTR Variant (NCT06563895) will include asymptomatic carriers of multiple variant forms of hATTR. It is a prospective, multi-center, double-blind and placebo-controlled study to evaluate the efficacy of acoramidis in asymptomatic carriers of a pathogenic TTR variant. Patients will be randomized to receive either acoramidis or placebo and the primary endpoint will measure time to development of hATTR-CM or hATTR polyneuropathy (hATTR-PN).

### Tolcapone

Tolcapone is a catechol-O-methyltransferase inhibitor. Although this was traditionally used for the treatment of Parkinson’s disease [34], it is also thought to have a high affinity for the T4 binding site. In vitro studies have demonstrated tolcapone disrupting fibril activity, which may in turn lead to an improvement in disease and reduction of amyloid deposition [35]. In the phase IIa clinical trial to demonstrate proof of concept, 17 subjects had ATTR with 55% of them having cardiac involvement, and there was a notable increase in TTR stabilization in plasma associated with tolcapone after 15 weeks of follow up [36]. Further studies are needed to establish the efficacy and utility of tolcapone as a TTR stabilizer.

## TTR Silencers

Transthyretin silencers reduce intrahepatic TTR production by preventing the transcription of its messenger RNA (mRNA). They have three different mechanisms: (1) small interfering RNA agents, which include patisiran, revusiran, and vutrisiran; (2) anti-sense oligonucleotides, which include inotersen and eplontersen; (3) gene editing (Table 2).

**Table 2:** TTR silencing therapies for transthyretin amyloid cardiomyopathy (ATTR-CM)

Drug	Clinical trial (year)	Design	Population	% Black patients	V142I patients	Outcomes
Small Interfering RNA agents						
Patisiran* (formerly known as ALN-TTR02)	APOLLO-B [16]	Phase 3, multicenter randomized, double-blind, placebo-controlled clinical trial for 18 months to receive patisiran or placebo	*N* = 360 (hATTR-CM, *n* = 72)	8.6% (*n* = 31)	40.3% (*n* = 29)	Lower decline in 6MWT distance, increased KCCQ-OS score, more infusion-related reactions, arthralgia, muscle spasms associated with patients in patisiran group
Revusiran* (formerly known as ALN-TTRsc)	ENDEAVOUR [38]	Phase 3, randomized, 2:1 ratio, double-blind, placebo controlled multicenter clinical trial for 18 months	*N* = 206 (hATTR-CM)	51% (*n* = 104)	57% (*n* = 117)	Trial terminated early due to increased mortality compared to placebo
Vutrisiran* (formerly known as ALN-TTRsc02)	HELIOS-B [18]	Phase 3, randomized, double-blind, placebo controlled multicenter clinical trial received SC vutrisiran 25 mg every 3 months for 36 months	*N* = 655 (hATTR-CM, *n* = 76)	7.2% (*n* = 47)	64% (*n* = 49)	Lower all-cause mortality and recurrent CV events, 0.72; change from baseline to month 30 in 6-MWT distance, KCCQ-OS favoring vutrisiran
Antisense Oligonucleotides						
Inotersen*	NEURO-TTR [41]	Phase 3, randomized, 2:1 ratio, double-blind, placebo controlled clinical trial to receive weekly SC inotersen (300 mg) or placebo over 15 months	*N* = 172 (hATTR-PN; 63%, *n* = 108, subset with cardiomyopathy)	2.3% (*n* = 4)	Not reported (52%, *n* = 89) Val30Met – associated with more polyneuropathy symptoms)	Change from baseline in mNIS + 7, QOL-DN, favoring inotersen
	(cardiac subgroup) [44]	Single-center, open-label, protocol over 3 years	*N* = 33 (*n* = 10 hATTR-CM)	Not reported	Not reported	Decrease in mean LV mass by 8.4% / 11.4% on MRI and increased 6MWT distance by 20.2 / 16.2 m in years 2 and 3, respectively
Eplontersen* (formerly known as ION-682884, IONIS-TTR-LRx, AKCEA-TTR-LRx)	CARDIO-TTRansform (2025, ongoing)	Phase 3 multicenter, randomized, double-blind, placebo-controlled clinical trial to receive subcutaneous injections of eplontersen or placebo every 4 weeks	Target enrollment of 1400 patients with hATTR-CM or wtATTR-CM, thus far *N* = 1438 patients enrolled	Not reported	Not reported	Pending
Gene Editing (CRISPR-Cas9)						
NTLA-2001*	MAGNITUDE (2028, ongoing)	Phase 3 multicenter randomized, 2:1 ratio double-blind, placebo-controlled study to receive a single 55 mg infusion of NTLA-2001 or placebo	Target enrollment of 765 patients with hATTR-CM or wtATTR-CM enrolled	Not reported	Not reported	Lower composite endpoint of cardiovascular-related (CV) mortality and CV-related events

(Table 2 partially adapted from: Tomasoni D, et al. Front. Cardiovasc. Med., vol. 10, May 2023:10:1154594. 10.3389/fcvm.2023.1154594; Creative Commons user license https://creativecommons.org/licenses/by/4.0/) [2]

* Inclusion criteria: patients aged 18–90 diagnosed with hereditary or wild type transthyretin amyloidosis confirmed by biopsy (e.g. endomyocardial, abdominal fat pad, gastrointestinal, bone marrow, tissue), with mass spectrometry or immmunohistochemical analysis or scintigraphy and biochemical analysis for exclusion of light chain (AL) amyloidosis

### Patisiran

Patisiran, formerly known as ALN-TTR02, is an siRNA with a lipid nanoparticle delivery system which targets hepatocytes at the common 3’ untranslated region of the TTR-mRNA and mediates the cleavage of TTR-mRNA to prevent expression which ultimately inhibiting the production of hepatic transthyretin in both hATTR and wtATTR [16, 37]. It is injected intravenously every 3 weeks. It was FDA approved in 2018 for treatment of patients with hATTR-PN [38].

A Study to Evaluate Patisiran in Participants With Transthyretin Amyloidosis With Cardiomyopathy (APOLLO-B; NCT03997383) was a phase 3, double-blinded, randomized clinical trial which enrolled 360 patients with hATTR or wtATTR in a 1:1 ratio to receive patisiran (181 patients received 0.3 mg/kg body weight) or placebo (179 patients) [16]. The primary endpoint was a change from baseline in the distance covered on the 6MWT and Kansas City Cardiomyopathy Questionnaire Overall Summary Score (KCCQ-OS) at 12 months. The trial demonstrated less decline in 6MWT distance and higher KCCQ-OS score in the patisiran group compared to placebo, indicating preserved functional capacity in patients with ATTR-CM [16]. The effects of patisiran were also notable on CMR. Fontana et al. examined the effect of patisiran on cardiac amyloid load as noted by CMR and extracellular volume (ECV) mapping in 16 patients with ATTR-CM [37]. A total of 82% of cases showed > 80% knockdown. Patisiran therapy was associated with reduced ECV, reduced cardiac uptake by bone scintigraphy, fall in NT-proBNP, and increased 6MWT distances [37].

Despite these findings, patisiran was never approved for cardiomyopathy and is currently indicated only for treatment of polyneuropathy in patients diagnosed with hATTR [38].

### Revusiran

Revusiran, formerly known as ALN-TTRsc, is another siRNA therapeutic agent which is conjugated to a triantennary *N*-acetylgalactosamine (GalNAc) ligand for delivery to the liver [39, 40]. This type of delivery system limits its metabolic stability compared to patisiran, hence requiring weekly treatment at relatively higher doses. Like patisiran, it also targets a region of TTR-mRNA that is common in to both the genetic variants of hATTR and wtATTR.

The Phase 3 Multicenter Study of Revusiran in Patients With Transthyretin Mediated Familial Amyloidotic Cardiomyopathy (ENDEAVOUR; NCT02319005) was a multicenter, placebo-controlled, double-blinded study of patients with hATTR amyloidosis with evidence of cardiomyopathy who were randomized in a 2:1 ratio to receive subcutaneous daily revusiran 500 mg (140 patients) or placebo (66 patients) for 5 days over a week followed by weekly dosing [39]. However, this trial was terminated early, after a median of 6.71 months, due to the concerns for new-onset or worsening peripheral neuropathy in some participants in the concurrently running Phase 2 OLE. While this investigation did not identify any concerns related to peripheral neuropathy, there was an imbalance in mortality in the revusiran arm compared to placebo, therefore the trial was discontinued [39]. Non-clinical safety profile conducted by Sutherland et al. was published after the early termination of the trial [40]. These safety assessments included safety pharmacology, acute and repeat-dose toxicity, genotoxicity, and carcinogenicity. There were no effectives on cardiovascular or respiratory function in monkeys after single doses up to 100 mg/kg nor any neurological effects in repeat-dose studies up to 300 mg/kg [40]. It was found to be well-tolerated in weekly repeat dose mouse studies and rat and monkey (5 daily doses followed by weekly doses) toxicity studies. Dose-limiting toxicity in monkeys was absent with the no observed adverse effect level (NOAEL) of 200 mg/kg [40]. Overall, the results supported a favorable non-clinical safety profile.

A crucial point of this study compared to other clinical trials of disease-modifying therapies in ATTR-CM is the inclusion of high-risk patients who were sicker at baseline. The study included equal enrollment of Black patients (51%) with a sizable representation of the V142I-variant of hATTR-CM (57%) [39]. Epidemiological studies extrapolate that as many as 1 in 20 Black patients have the V142I variant (approximately 3–4% of the Black population in the United States [5, 41]. They are more likely to have delays in diagnosis, more likely to develop the later effects of cardiomyopathy and subsequent heart failure [5, 41]. It is possible that a portion of the imbalance in mortality seen in the phase 3 clinical trial of revusiran may be related to the higher representation of Black patients, who tend to be sicker at baseline prior to clinical trial enrollment. Future clinical trials of ATTR-CM disease modifying therapies have failed to include equitable representation of Black patients and especially those with V142I genetic variant.

### Vutrisiran

Vutrisiran, formerly known as ALN-TTRsc02, is an agent in the siRNA class of ATTR therapeutics [17, 18]. Similar to revusiran, but in contrast to patisiran’s lipid nanoparticle delivery system, it uses the GalNAc conjugate delivery system.

A Study of Vutrisiran in Patients With Hereditary Transthyretin Amyloidosis (HELIOS-A; NCT03759379) was a global, open-label, phase 3, double-blinded clinical trial of the FDA approved patisiran versus vutrisiran [17]. In this trial, 164 patients with hATTR were randomized in a 3:1 ratio to subcutaneous vutrisiran 25 mg every 3 weeks (122 patients) or intravenous patisiran 0.3 mg/kg every 3 weeks (42 patients) with a placebo group (77 patients) as [18]modified Neuropathy Impairment Score + 7 (mNIS + 7) at 9 months. The study determined the reduction in serum TTR in the vutrisiran arm was non-inferior to patisiran. There were no drug-related discontinuations or deaths. It was approved by the FDA in June 2022 and the EMA in July 2022 as the 2nd siRNA therapeutic agent for the treatment of Stage 1 or Stage 2 polyneuropathy in patient with hATTR amyloidosis.

A Study to Evaluate Vutrisiran in Patients with Transthyretin Amyloidosis With Cardiomyopathy (HELIOS-B; NCT04153149) was an international, multicenter, phase 3, open-label extension, double-blinded, randomized trial. Fontana et al. investigated vutrisiran for the treatment of cardiomyopathy in patients with hATTR [18]. In this study, 655 patients were treated with subcutaneous vutrisiran quarterly and demonstrated a 28% lower risk of all-cause mortality and recurrent cardiovascular events compared to patients in the placebo arm at 36 months. For the 395 patients on vutrisiran monotherapy (not on tafamidis at baseline) had a 33% lower risk of all-cause mortality. In the overall population, treatment with vutrisiran also showed less of a decline in the distance covered by 6MWT and KCCQ-OS score. It is important to note, there was substantial use of effective standard care therapies for ATTR-CM and heart failure. These included tafamidis (baseline ~ 40% in both arms), sodium-glucose cotransporter-2 inhibitors (baseline ~ 3% in both arms), and diuretics (baseline ~ 80% in both arms). Patient were not randomized to baseline tafamidis and those on this TTR stabilizer were generally healthier based on NYHA class, NT-proBNP, 6-MWT distance, and KCCQ-OS score. The safety profile analysis showed vutrisiran was well-tolerated with the majority of adverse events categorized as either mild or moderate and with no statistically different serious adverse events seen in the vutrisiran arm compared to the placebo arm. There were more deaths in the placebo group (19.2%, *n* = 63) compared to the vutrisiran group (15%, *n* = 49). The results from recent ground-breaking trial may pave the way for another effective therapy on the market soon.

### Inotersen

Inotersen is an antisense oligonucleotide that binds to TTR-mRNA to increase the degradation of both variant and wild-type transthyretin mRNA which reduces the TTR protein in serum and tissue [1, 3, 4, 42–45].

The Efficacy and Safety of Inotersen in Familial Amyloid Polyneuropathy (NEURO-TTR; NCT01737398) was a phase 3 clinical trial of inotersen in 172 adults with stage 1 (patient is ambulatory) or stage 2 (patient is ambulatory with assistance) were randomized in a 2:1 ratio to receive weekly subcutaneous inotersen 300 mg (112 patients) or placebo (60 patients) [42]. There were three primary endpoints: (1) serum transthyretin concentration; (2) change in mNIS + 7 score, with higher scores indicating poorer function; (2) change in the score of patient-reported Norfolk Quality of Life Diabetic Neuropathy (QOL-DN) questionnaire with higher scores indicating poorer quality of life. Both primary endpoints demonstrated statistically significant change from baseline for patients treated with inotersen [42]. There were five deaths in the inotersen arm and none in the placebo arm with the most serious adverse events were glomerulonephritis (3 patients) and thrombocytopenia (grade 4 thrombocytopenia associated with 1 of the 5 deaths). 97% of patients who completed NEURO-TTR trial (*n* = 1390 were enrolled and completed its open-label extension trial [43]. No new safety concerns were identified and there no evidence of increased risk of renal or hematologic events with increased duration of inotersen exposure. In the follow-up of the open-label extension trial, inotersen treatment for more than 3 years slowed progression of polyneuropathy associated with hATTR and there were no additional safety signals found [44]. The subcutaneous formulation of inotersen (284 mg) was approved by EMA on July 6, 2018, and later by the FDA on October 5, 2018, for the treatment of Stage 1 and Stage 2 polyneuropathy of patients with hATTR [45].

There was a small, single-center, open-label study of inotersen in 33 patients with hATTR-CM or wtATTR-CM [46]. It demonstrated decreased LV mass by 8.4% (measured on CMR), and increased 6MWT distance both at year 2 and year 3. However, inotersen was not comprehensively studied further or currently approved for ATTR-CM.

### Eplontersen

Eplontersen, formerly known as ION-682,884, IONIS-TTR-LRx, and AKCEA-TTR-LRx, is another antisense oligonucleotide that binds to TTR-mRNA to promote degradation of the subsequent TTR-mRNA product to reduce the serum TTR protein and its deposition in tissue [47].

A Study to Evaluate the Efficacy and Safety of Eplontersen in Patients With Hereditary Transthyretin-Mediated Amyloid Polyneuropathy (NEURO-TTRansform; NCT04136184) was an open-label, single-group, phase 3 trial conducted at 40 sites in 15 countries of 168 adults with Stage 1 or 2 polyneuropathy in patients with hATTR who were treated with subcutaneous eplontersen 45 mg every 4 weeks (144 patients) or 300 mg weekly (24 patients) treated from December 2019 to April 2023 compared to the placebo arm (66 patients) from March 2013 to November 2017 [47]. The same primary endpoints used in the NEURO-TTR trial for inotersen were used in this trial: serum transthyretin concentration, change in mNIS + 7 score and change in QOL-DN score were used in this trial. There was an −70.4% reduction in serum transthyretin concentration in the eplontersen arm compared to the placebo arm. In total, there were 6 versus 2 discontinuation events in the eplontersen group compared to placebo; there were 2 deaths in the eplontersen group which was consistent with known disease-related sequelae (cardiac arrhythmia, intracranial hemorrhage). The FDA approved the use of eplontersen for treatment of polyneuropathy in patients with hATTR on December 21, 2023 [48].

A Study to Evaluate the Efficacy and Safety of Eplontersen in Patients With Transthyretin-Mediated Amyloid Cardiomyopathy (CARDIO-TTRransform; NCT04136171) is a counterpart trial that is investigating eplontersen for cardiomyopathy in hATTR patients. It is a phase 3 clinical trial which enrolled more than 1,400 patients making it one of the largest studies in this patient population to date and projected to share data in 2025.

### NTLA-2001

NTLA-2001 is an in vivo gene-editing therapy designed to reduce TTR in serum using the technology based on clustered regularly interspaced short palindromic repeats and associated Cas9 endonuclease (CRISPR-Cas9) system [20]. It includes mRNA for Cas9 protein and a single guide RNA targeting the TTR protein encapsulated in a lipid nanoparticle coat.

Preclinical in vitro and in vivo studies using mice and monkeys showed durable knockout of TTR after a single dose [20, 21].

A Study to Evaluate Safety, Tolerability, Pharmacokinetics, and Pharmacodynamics of NTLA-2001 in Patients With Hereditary Transthyretin Amyloidosis with Polyneuropathy and Patients With Transthyretin Amyloidosis-Related Cardiomyopathy with a Phase 1 clinical trial with two parts: Part 1 (open-label, single ascending dose) and Part 2 (open-label, single dose expansion) (NCT04601051). In published results from Part 1, Gilmore *et*. *al*. evaluated the safety and effect of single escalating doses of NTLA-2001 in 6 patients with hATTR-PN with three patients in each of the initial groups (0.1 mg/kg and 0.3 mg/kg) as part of the ongoing trial [20]. They demonstrated a decrease in serum TTR protein concentrations due to target knockout of TTR with minimal side effects. The follow-up investigation of the 72 patients enrolled in the Phase 1 trial showed that there are 15 out of 36 patients with hATTR-PN and 12 out of 36 patients with hATTR-CM in Part 1: single-ascending dose escalation [49]. There were 16 out of 21 patients with hATTR-PN who received the 55 mg dose compared to the 80 mg and 55 mg in 24 patients with hATTR-CM with NYHA Class I/II versus Class III in Part 2: dose expansion [49]. After completing the protocol-specific, 2-year observation period, patients who received a single dose of 55 mg had consistently low and sustained absolute serum TTR concentrations with over 80% knockdown and was well-tolerated [49].

A Phase 3 Study of NTLA-2001 in Participants With Transthyretin Amyloidosis With Cardiomyopathy (MAGNITUDE; NCT06128629) is a multicenter, quadruple-blind, placebo-controlled clinical trial in which an estimated 765 patients will be randomized in a 2:1 ratio to receive a single intravenous infusion of 55 mg of NTLA-2001 or placebo and follow-up for a minimum of 18 months and up to 48 months. The primary endpoint is a composite outcome of cardiovascular mortality and frequency of CV-related events. Secondary outcomes are change from baseline to month 18 in serum TTR concentrations and KCCQ-OS score. The anticipated study completion will be 2028.

## TTR Depleters

The therapeutic approach for ATTR-CM is evolving, with TTR depleters emerging as a promising class of agents targeting the pathological accumulation of amyloid deposits using immunotherapy, particularly monoclonal antibodies (Table 3). These therapies work by promoting antibody-mediated clearance of amyloid deposits, with antibodies tagging the deposits for removal by the immune system, primarily through macrophages. This process aims to deplete existing amyloid deposits in tissues, thereby mitigating disease progression and improving cardiac function. Below, we discuss the key TTR depleters currently under investigation.

**Table 3:** TTR depleting agents for transthyretin amyloid cardiomyopathy (ATTR-CM)

Name	Type	Target mechanism	Current phase	Infusion details
Monoclonal antibodies				
NNC6019-0001* (formerly known as PRX004)	IgG1	A humanized monoclonal antibody targeting misfolded and aggregated forms of TTR.	Phase 2 (NCT05442047)	Participants received intravenous (IV) infusion of either 10 mg/kg or 60 mg/kg NNC6019-0001 or placebo every 4 weeks, added to standard care until week 52
ALXN2220* (formerly known as NI006)	IgG1	Recombinant human IgG1 monoclonal antibody targeting amyloid conformations of TTR. Developed from memory B cells from healthy older individuals.	Phase 3 (DepleTTR-CM, NCT06183931)	Participants received weight based IV infusion of ALXN2220 or placebo every 4 weeks for 24–48 months.
AT-02	IgG1-Peptide Fusion	Fusion of IgG1 with a peptide (p5R) with pan-amyloid reactivity. Targets amyloid fibrils through electrostatic interactions.	Preclinical	N/A

* Inclusion criteria: patients aged 18–90 diagnosed with hereditary or wild type transthyretin amyloidosis confirmed by biopsy (e.g. endomyocardial, abdominal fat pad, gastrointestinal, bone marrow, tissue), with mass spectrometry or immmunohistochemical analysis or scintigraphy and biochemical analysis for exclusion of light chain (AL) amyloidosis

### NNC6019-0001

NNC6019-0001, previously known as PRX004, is a humanized monoclonal IgG1 antibody that targets a specific epitope of TTR, which is exposed on misfolded monomeric and aggregated forms of TTR but remains hidden in native tetramers [50]. In a phase 1 study involving patients with hATTR-CM, NNC6019-0001 demonstrated promising results. Neurological improvements were observed, with a mean change in mNIS + 7 of −3.33 points in some patients, and cardiac systolic function, measured by Global Longitudinal Strain (GLS), also showed improvement [51]. The phase 2 clinical trial (NCT05442047) is currently ongoing [19]. These early results suggest that NNC6019-0001 could be a viable option for slowing disease progression in ATTR amyloidosis​.

### ALXN2220

ALXN2220, previously known as NI006, is a recombinant human IgG1 monoclonal antibody designed to target amyloid conformations of both wtATTR and hATTR. It was developed by analyzing the immune repertoire of memory B cells from healthy older individuals. This antibody specifically binds to the amyloid forms of TTR while avoiding the physiologically folded tetrameric TTR structure [52]. Preclinical studies demonstrated that ALXN2220 could deplete ATTR deposits through antibody-mediated phagocytosis, effectively removing amyloid from cardiac tissues [53]. The safety and efficacy of ALXN2220 was evaluated in a phase 1 clinical trial (NCT04360434) in patients with wild-type or variant ATTR cardiomyopathy. The study reported no serious drug-related adverse events, and imaging studies indicated a reduction in cardiac amyloid load over 12 months [53].

The Study of ALXN2220 Versus Placebo in Adults With ATTR-CM (DepleTTR-CM; NCT06183931) is a large-scale phase 3 clinical trial that is now enrolling patients to further investigate the efficacy of this antibody in patients with ATTR-CM with results expected to provide more definitive evidence of its therapeutic potential​.

### AT-02 (Attalus)

AT-02 represents a novel approach in TTR depletion therapy, leveraging a fusion of IgG1 with a peptide that has pan-amyloid reactivity. The peptide component, p5R, binds to amyloid fibrils through electrostatic interactions, while the IgG1 component facilitates clearance [54]. In preclinical studies, AT-02 demonstrated rapid and specific binding to amyloid deposits in various organs, including the liver, spleen, and heart. It showed the capability to persist within amyloid deposits for extended periods, enabling continuous clearance. This novel agent holds promise as a broad-spectrum amyloid depleter, potentially applicable to various types of amyloidosis, including ATTR​ [55].

TTR depleters represent a critical advancement in the treatment of transthyretin cardiac amyloidosis. These therapies are still in the experimental stages, but early results indicate their potential to significantly reduce amyloid burden and improve clinical outcomes. Continued research and clinical trials will be essential to establish their efficacy and safety, paving the way for their integration into standard treatment protocols for ATTR amyloidosis.

### Doxycycline + Tauroursodeoxycholic / Ursodeoxycholic Acid

In preclinical studies, doxycycline, which is a tetracycline antibiotic [56], and tauroursodeoxycholic acid (TUDCA), a type of naturally occurring hydrophilic bile acid [57], has been shown to reduce TTR amyloid deposits. The combination of these therapies was found to have synergistic effects in familial amyloidotic polyneuropathy (FAP) transgenic mice models [58]. Since these drugs degrade amyloid fibrils, they can theoretically target the disease later in its course compared to ATTR stabilizers and silencers.

Early clinical data using this combination of drugs was derived from a small open-label, phase 2 study in which 20 patients (*n* = 17 with FAP, *n* = 2 with senile systemic amyloidosis, and *n* = 1 who was a domino liver transplant recipient) were given doxycycline 100 mg twice daily and TUDCA 250 mg three times daily for 12 months [59]. The study found no progression of polyneuropathy or cardiac involvement. However, two patients discontinued the treatment due to gastric pain and nausea with anorexia.

A retrospective study of 53 patients with ATTR-CM studied the effects of doxycycline and ursodeoxycholic acid (UDCA), also known as ursodiol, another form of bile acid [60]. Six patients did not tolerate treatment due to gastrointestinal side effects and photosensitivity. Of the remaining 47 patients there were no signs of disease progression for the majority of participants.

An open-label, phase 3, randomized clinical trial (NCT03481972) investigated the combination of these two drugs in 102 patients with ATTR-CM. The trial was designed with patients enrolled prior to the approval of tafamidis for ATTR-CM. The primary endpoint was the survival at 18 months and demonstrated that the combination had no impact on survival and doxycycline has no role in treatment for ATTR (data unpublished).

### Additional Therapies / Nutritional Supplements

Both turmeric and epigallocatechin-3-gallate (EGCG) have been studied as possible treatments for ATTR. While they have not been approved as effective therapies, they are readily available over-the-counter and are used by patients as additive interventions to standard of care therapies.

### Turmeric

Curcumin, the active polyphenol in turmeric, has been considered as a possible treatment for ATTR. One in vitro study, demonstrated that physiologically achievable doses of curcumin strongly reduce TTR-aggregates-induced endoplasmic reticulum (ER) stress response and apoptosis [61]. It also showed suppression of TTR fibril formation via stabilization of the TTR tetramer or by formation of nontoxic aggregates.

Furthermore, Ferreira et al. investigated the effects of dietary curcumin supplementation in family amyloidotic polyneuropathy (FAP) mouse models. It showed that curcumin selectively binds to TTR thyroxine binding sites, resulting in a more stable TTR tetramer. Additionally, it reduced TTR deposition by up to 70% in some tissues and decreased activation of its associated biomarker. Looking at a wide range of concentrations (0.01–100 µM), they found the most optimal dose of curcumin is 1 µM, with no increased aggregate internalization by macrophages at higher concentrations [61].

### Epigallocatechin Gallate

EGCG is a catechin with the highest concentrations found in green tea. In vitro studies have demonstrated that ECCG can inhibit formation of amyloid fibrils. A small observational study of 19 patients with ATTR-CM were evaluated by lab results, echocardiography, and CMR (*n* = 9) before and after consumption of green tea and/or green tea extracts [62]. After 12 months of follow-up, there was no increased LV wall thickness or LV myocardial mass by echocardiography. In the subset of patients evaluated by CMR, there was a mean decrease in LV myocardial mass (−12.5%), suggesting an inhibitory effect of green tea/extract, which slowed the progression of ATTR-CM. However, a retrospective study by Capell et al. with a larger sample of 65 patients with a 675 mg daily dose of EGCG compared to placebo over a minimum of 9 months of follow-up did not show EGCG having any mortality benefit albeit without major safety concerns [63]. It is considered a safe therapeutic option. However, there is no association with increased survival.

## Conclusions

Increased awareness of the prevalence and epidemiology of ATTR-CM, earlier diagnosis with imaging have paved the way for the development and use of several of these multi-target therapies. Previously, tafamidis was the only approved treatment for ATTR-CM, but with recent success and ongoing clinical trials for novel therapies, we expect the landscape for treatments to expand. As more treatments are available, efforts will need to refocus on understanding long-term effects and improving reach and access for the most vulnerable populations. We must intertwine in these missions: increased diversity in clinical trial enrollment, as well as develop affordable pathways to allow equitable access to ground-breaking therapies.

## Key References

- Fontana M, Berk JL, Gillmore JD, Witteles RM, Grogan M, Drachman B, Damy, T et al. Vutrisiran in Patients with Transthyretin Amyloidosis with Cardiomyopathy. N Engl J Med. 2024 Aug 30.
  - Findings from this recent study suggest that a TTR silencer lowers all-cause mortality and frequency of CV events, increasing the possibility of another agent approved for treatment of transthyretin cardiomyopathy.
- Gillmore JD, Judge DP, Cappelli F, Fontana M, Garcia-Pavia, P et al. Efficacy and Safety of Acoramidis in Transthyretin Amyloid Cardiomyopathy. N Engl J Med. 2024.
  - Findings from this recent study demonstrate a second TTR stabilizer agent that may be approved for treatment of transthyretin cardiomyopathy in the next few years.
- Gillmore JD, Gane E, Taubel J, Kao J, Fontana M, et al. CRISPR-Cas9 In Vivo Gene Editing for Transthyretin Amyloidosis. N Engl J Med. 2021; 385(6): 493–502.
  - Findings from this study demonstrate the efficacy of a single infusion of a gene-editing therapy that can significantly decrease serum TTR protein concentrations. The therapy is currently being studied in a Phase 3 clinical trial.

## Data Availability

No datasets were generated or analysed during the current study.
